# Canned Fish in Brine—Variability in Macronutrient and Fatty Acid Composition

**DOI:** 10.3390/biology15050381

**Published:** 2026-02-26

**Authors:** Diana Chrpová, Vojtech Ilko, Markéta Růžičková, Miroslava Potůčková, Lenka Kouřimská, Pavel Kohout, Jan Pánek, Marek Doležal

**Affiliations:** 1 Department of Microbiology, Nutrition and Dietetics, Czech University of Life Sciences Prague, Kamýcká 129, 165 00 Prague, Czech Republic; chrpovad@af.czu.cz (D.C.); kourimska@af.czu.cz (L.K.); 2 Department of Food Analysis and Nutrition, University of Chemistry and Technology, Technická 5, 166 28 Prague, Czech Republic; vojtech.ilko@vscht.cz (V.I.);; 3 4th Department of Internal Medicine, 1st Faculty of Medicine, Charles University and General University Hospital in Prague, U Nemocnice 2, 128 08 Prague, Czech Republic; maky.ruzickova@gmail.com; 4 Department of Dairy, Fat and Cosmetics, University of Chemistry and Technology, Prague, Technická 5, 166 28 Prague, Czech Republic; mirka.mihulova@seznam.cz; 5 Department of Internal Medicine, 3rd Faculty of Medicine, Charles University and Thomayer University Hospital, Ruská 87, 100 00 Prague, Czech Republic; pavel.kohout@lf3.cuni.cz

**Keywords:** protein, lipids, fat, EPA, DHA, dry matter

## Abstract

Marine fish are a good dietary source of important macro- and micronutrients. In addition to fresh fish, fish with varying degrees of industrial processing—frozen, marinated, smoked, canned, etc.—is used extensively in the food market. This study comprehensively characterizes the protein content, fat content, and fatty acid composition of various commercially available canned marine fish species. All canned fish muscle and cod liver were in salted brine, to eliminate the influence of other ingredients. All samples obtained from muscle had a relatively high protein content, mostly between 15 and 21 g/100 g. The fat content was highly variable, ranging from a few tenths to 15 g/100 g. Of the fatty acids, PUFAs predominated in almost all samples. The fatty acid composition of canned fish was very similar to the fatty acid composition of fresh fish. The fact that the content of highly oxylabile PUFAs practically did not differ compared to fresh fish indicates that the preservation process does not lead to significant oxidative damage to this type of product. A significant benefit of eating marine fish is the intake of highly unsaturated fatty acids EPA and DHA of the omega-3 fatty acid series. Mainly mackerel and sardines are an excellent source of these two acids. Atlantic salmon and sockeye salmon are also very good sources. As a result, consuming an average of 3 g of cod liver, 10 g of mackerel, 15 g of sardines, or 30 g of Atlantic and sockeye salmon is sufficient to ensure the recommended daily intake of EPA and DHA.

## Simple Summary

Marine fish are an important source of high-quality protein and beneficial fats in the human diet. In addition to fresh fish, canned fish is also widely consumed, but information on its nutritional value is limited. This study examined the protein, fat, and fatty acid composition of various commercially available canned fish species in brine. All samples contained relatively high amounts of protein, generally between 15 and 21 g per 100 g, while fat content varied from a few tenths to 15 g per 100 g, depending on the fish species. Canned marine fish proved to be a valuable source of the omega-3 polyunsaturated fatty acids, in particular eicosapentaenoic and docosahexaenoic acid, which are important for human health. As a result, consuming an average of 3 g of cod liver, 10 g of mackerel, 15 g of sardines, or 30 g of Atlantic and sockeye salmon is sufficient to ensure the recommended daily intake of these two acids. However, it would be necessary to consume 300–700 g of skipjack tuna. On the other hand, tuna remains a source of high-quality protein and other nutritionally valuable substances.

## 1. Introduction

From a nutritional point of view, fish are primarily a source of high-quality protein, some minerals, and important micronutrients. Some fish (especially fatty fish—salmon, herring, sardines, anchovies, mackerel, etc.) are very good sources of polyunsaturated fatty acids (PUFAs), in particular eicosapentaenoic acid (EPA) and docosahexaenoic acid (DHA). For fatty fish, the usable energy is mainly due to the fat content, while for other fish it is mainly due to the protein content [1,2,3,4,5].

The protein content of fish is usually in the range of 16–22 g/100 g. Muscle protein has a high nutritional value and is very easily digestible. Fish are also an important dietary source of some non-proteinogenic amino acids with high biological activity, e.g., carnitine or creatine [1,3,4,5,6,7,8,9,10].

The fat content in fish muscle is very variable, ranging from tenths of a gram to 25 g in 100 g of product. The content of saturated fatty acids (with predominant palmitic acid) ranges from 15 to 30% of the fat content. The presence of omega-3 PUFAs with a long chain and a higher number of double bonds, so-called HUFAs (highly unsaturated fatty acids), is significant. They are not synthesized in plants (except for genetically modified) and are found in significant quantities only in fish and seaweed. Freshwater fish contain lower levels of omega-3 HUFA. Of marine fish, only those that contain a larger amount of fat are a good source of EPA and DHA. They include, for example, Atlantic salmon (*Salmo salar* L.), Atlantic mackerel (*Scomber scombrus* L.), Atlantic herring (*Clupea harengus* L.), sardine (*Sardina pilchardus* Walb.), bluefin tuna (*Thunnus thynnus* L.) or swordfish (*Xiphias gladius* L.). To ensure the recommended daily intake of 250 mg EPA + DHA, 10–20 g of any of these fish is sufficient [1,3,4,5,6,7,9,11,12,13,14,15,16,17,18,19].

Canning and freezing technology are widely used in fish processing. In both cases, the technology must be conducted in such a way as to prevent, if possible, the loss of significant, chemically unstable nutrients. In the case of fish, these are mainly oxylabile HUFAs. In the case of fish products, the problem of oxidation can be solved by a suitable technological procedure or by the addition of effective natural antioxidants, e.g., rosemary extracts [3,9,12,16,20,21,22,23,24,25,26].

Even experts can find it difficult to follow the nomenclature of certain fish species. Identifying fish processed into various fish products then becomes a significant problem. A good example is the nomenclature of fish referred to by the English name tuna. This group includes four genera (*Auxis*, *Euthynnus*, *Katsuwonus* and *Thunnus*), all belonging to the *Scombridae* family, with a total of 13 species. These species differ significantly in composition; e.g., the fat content ranges from 0.5 to 16 g/100 g [27,28]. The aim of this study was therefore to comprehensively characterize macronutrient levels of various commercially available canned fish species that are currently produced worldwide and widely available on the European Union food market. This study is of major importance for public health, accurate dietary recommendations, food industry standards, and consumer awareness.

## 2. Materials and Methods

Samples of canned fish and fish products in brine (various producers and various batches; a total of 46 samples) were purchased for laboratory testing in 2024 in Czech Republic markets. All samples were at least 6 months prior to the best-before date at the time of analysis. In the cod liver (CL) samples, fish liver was processed; in other samples, the processed part was muscle tissue. Samples of canned fish in some vegetable oils or tomato sauce were not considered, because these ingredients could affect the fatty acid profile of the tested fish. Table 1 summarizes the basic characteristics of the analyzed samples.

For each sample, three cans were opened, and the entire contents of the drained solid portion were homogenized using a flesh-suitable mixer, Grindomix GM200 (Retsch GmbH, Haan, Germany), and kept in the freezer at −55 °C until the analysis.

Dry matter was determined gravimetrically after drying approximately 1.5 g of homogenized samples at 105 °C in a laboratory drying oven, Binder E28 (Binder GmbH, Tuttlingen, Germany), to a constant weight according to ISO1442:2023 [29]. The Kjeldahl method was used to determine nitrogen by the KT200 Kjeltec system (FOSS, Hillerød, Denmark) according to AOAC method 920.105 [30]. The protein content was estimated by multiplying the determined nitrogen content by a nitrogen-to-protein conversion factor of 6.25 [31]. The fat content was determined gravimetrically after extraction according to ISO 659:2009 [32], adapted for the Soxtec 8000 apparatus (FOSS, Hillerød, Denmark). Fatty acids were first converted to fatty acid methyl esters according to EN ISO 12966-2:2011 [33] and then analyzed by gas–liquid chromatography using an SP-2560 fused silica capillary column (100 m × 0.25 mm i.d., 20 μm film thickness) (Supelco, Bellefonte, PA, USA) in an Agilent 6890 gas chromatograph (Agilent Technologies, Santa Clara, CA, USA) equipped with a flame ionization detector (FID) under the conditions described by Sabolová et al. [34]. The fatty acid quantification was carried out by the internal normalization method, and the results were expressed as relative percentages of all identified fatty acids.

For all of the above measurements, two parallel determinations were performed for each sample. In order to verify the reliability of the results, six parallel determinations of the previously mentioned methods were performed for a selected sample. The repeatability, expressed as relative standard deviation (RSD), did not exceed 5% for any of the methods. The statistical analysis comprised one-way analysis of variance (ANOVA) and cluster analysis, which was performed using STATISTICA v. 12.0 (StatSoft, Inc., Tulsa, OK, USA). Sheffe’s post hoc test was performed at the 5% significance level to identify differences among tested samples. Partial least squares discriminant analysis (PLS-DA) was performed on the free online platform MetaboAnalyst 6.0 (Xia Lab, McGill University, Montreal, QC, Canada). No normalization, transformation, or scaling was used prior to the analysis.

## 3. Results

### 3.1. Dry Matter, Protein, and Fat Content

A total of 46 canned fish samples commonly available on the Czech market were analyzed for dry matter, protein, and fat content. Table 2 summarizes the results of the proximate composition of the tested samples.

Significant differences (*p* = 0.05) for all three parameters (dry matter, total fat, and crude protein contents) were found for all sample groups (T = tuna; CL = cod liver; M = mackerel; S = salmon; O = salmon nerka; SP = sardine). Within the individual groups, significant differences were not found for any parameter. The biggest differences can logically be observed in cod liver samples, where the processed part of the fish (liver) is different from the other samples, where the fish muscle is processed.

The protein content in fish samples (excluding cod liver) ranged between 15 and 22 g/100 g of sample. While the variability in protein content was relatively small in muscle fish samples, the variability in fat content was very high. Skipjack tuna had a very low amount of fat; all samples were found to contain less than 1 g/100 g. In the monitored samples of canned sockeye salmon, a fat content in the range of 2.8–4.3 g/100 g of the sample was found. A moderately high fat content was found in samples of mackerel, salmon (slightly above 10 g/100 g in both cases), and sardines (around 10 g/100 g on average).

### 3.2. Fatty Acid Composition

Fatty acid composition of the samples is summarized in Table 3 and Table 4.

**Figure 1:**
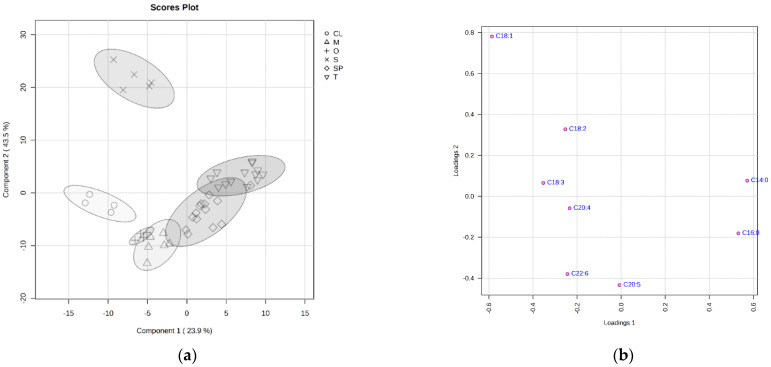
(**a**) PLS-DA score plot representing sample separation based on their fatty acid profile; sample groups: T = tuna (skipjack); CL = cod liver; M = mackerel; S = salmon; O = salmon nerka; SP = sardine samples. (**b**) PLS-DA loading plot complementary to the score plot (Figure 1a); fatty acid: C14:0 = myristic acid; C16:0 = palmitic acid; C18:1 = oleic acid; C18:2 = linoleic acid; C18:3 = α-linolenic acid; C20:5 = eicosapentaenoic acid (EPA); C22:6 = docosahexaenoic acid (DHA); C20:4 = arachidonic acid.

The contents of fatty acids in canned fish samples are expressed in Table 3 as the relative % of fatty acid from the total content of all fatty acids. Only selected fatty acids with a known physiological effect and oleic acid are displayed in the results [1,3,5,7,9,11,12,13,15,16,17,18,19,23,35,36]. The total contents of saturated acids (SFAs), monounsaturated acids (MUFAs), and PUFAs are also given (Table 4). The diversity of fatty acid profiles can be clearly demonstrated based on the PLS-DA results (Figure 1a,b).

It is interesting to monitor the distribution of MUFAs in the individual genera of the monitored fish. Oleic acid (C18:1, ∆9 *cis*) is the major acid in all cases. From wild fish, mackerel and sardine samples contain between 30 and 50% of the total MUFA content, sockeye salmon about 55%, and skipjack tuna about 66%. For farm-raised salmon, it is 74–80%. Of the other MUFAs, the most common are palmitoleic acid (C16:1, ∆9 *cis*) and the positional isomers of eicosenoic (C20:1) and docosenoic (C22:1) acid. They accounted for 20–70% of the total MUFA content.

The basic member of the group of omega-6 PUFAs is linoleic acid (C18:2, ∆9,12 *cis*, *cis*). The content of linoleic acid in wild fish is usually up to 3% of fatty acids; significantly more is contained in the fat content of the muscle of farm-raised salmon (12–15%), which is in good agreement with literary sources [1,15,18]. Biological activity is shown by arachidonic acid (C20:4, ∆5,8,11,14 all *cis*) from the group of omega-6 PUFAs. In most samples, its content was very low (up to 2%); the content was higher in mackerel and sockeye salmon samples (3–5%). In canned cod liver, 7.5–9.8% of arachidonic acid was found, which is the highest value of all fish tissue samples.

The basic member of the omega-3 PUFAs is α-linolenic acid (C18:3, ∆9,12,15 *cis*, *cis*, *cis*). The content of α-linolenic acid in wild fish is usually up to 2% of fatty acids; the content is higher in sockeye salmon samples (3.2–5.6%). More α-linolenic acid is also contained in the fat content of farmed salmon muscle (3.6–5.8%).

Cod liver had a very high fat content (55–60%), containing a small amount of saturated acids (around 20%) and, conversely, a large amount of PUFAs (44–53%), of which a significant proportion was α-linolenic acid (8.8–13.6%) and EPA and DHA (16.6–19.9%) (Table 3 and Table 4).

### 3.3. EPA and DHA Content

Undoubtedly, the most important source of EPA and DHA from the diet is marine fish from the cold sea with a higher fat content in the muscle tissue. Canned fish is also a very good source (see Table 3). Table 5 shows the amount of the product (canned fish) in which 250 mg of EPA and DHA are present.

From the results in Table 5, it can be seen that (excluding cod liver) the richest source of EPA and DHA is mackerel (less than 10 g of product is enough to receive 250 mg of EPA & DHA) and sardines (10–12 g of product). A very good source of EPA and DHA is also canned farm-raised Atlantic salmon and sockeye salmon (required intake of around 30 g per day). However, it can also be seen that in order to receive 250 mg of EPA and DHA, it would be necessary to consume 300–700 g of tuna. On the other hand, tuna remains a source of high-quality protein and other nutritionally valuable substances.

## 4. Discussion

As all samples were in brine, only a minimal amount of the other ingredients was added during their preservation. This, together with careful preservation, is the main reason why the data obtained, within the range of variability, are consistent with the data on fresh fish from databases and scientific articles [1,3,4,5,6,7,11,12,13,14,15,18,19,20,21,22,23,24,28].

In terms of the crude protein content, EFSA (2017) [2] reports a PRI value (Population Reference Intake) of 0.83 g/kg body weight/day for protein intake in an adult. At a weight of 70 kg, this corresponds approximately to the necessary intake of 58 g of proteins. Results in Table 2 show that one regular can of fish (usual weight 100–125 g) will supply a relatively significant proportion of the daily protein requirement. The obtained values of the fat content are also in very good agreement with published data. From a nutritional point of view, the amount of fat in the skipjack tuna sample can be considered completely insignificant. Fish of the genus *Oncorhynchus*—*O. tshawytscha* (Chinook salmon), *O. mykiss* (rainbow trout), *O. gorbuscha* (pink salmon), *O. nerka* (sockeye salmon), *O. kisutch* (coho salmon), and *O. keta* (chum salmon)—usually contain a small amount of fat, up to 5 g/100 g [1,4,5,6,7,11,12,13,15,18,19,20,28]. EFSA [2] recommends 20–35% of the total energy intake as an adequate fat intake. EFSA recommends an energy intake of 9.3 MJ/day for men and 7.5 MJ/day for women in middle age (40–49 years old) with moderate physical activity [2]. After conversion (useable energy from fat 37 kJ/g) [2], this recommendation corresponds to a fat intake of 50–88 g for men and 41–71 g for women. The results show that even the intake of medium-fatty fish, which included the case of sockeye salmon samples, is not the cause of a significant increase in fat intake in the diet.

The PLS analysis showed a significant difference through samples according to the fish species (Figure 1a,b). Salmon samples (S) were distinctly separated due to their higher proportion of oleic and linoleic acids resulting from farm feeding. Cod liver samples (CL) were also clearly separated by the higher proportion of linolenic and arachidonic acids as a result of metabolic processes different from those in muscle tissue. The fatty acid composition of the other samples was more similar, and therefore they were only partially separated. The determining fatty acids were DHA (predominant in mackerel and sockeye salmon samples) and EPA (in sardine samples). Tuna samples also contained these HUFAs; however, they were separated mainly due to the higher content of saturated acids (myristic and palmitic).

The evaluation of fatty acid composition (see Table 3 and Table 4) shows a significant share (71–88%) of the total content of saturated acids are atherogenic [17] myristic (C14:0) and palmitic (C16:0) acids. Skipjack tuna samples had a high proportion (around 50%) of saturated fatty acids. From a nutritional point of view, this fact is uninteresting, because the fat content here is extremely low (see Table 2). Mackerel, sockeye salmon, and sardines usually contain slightly more than 30% saturated acids, which is very acceptable from a nutritional point of view. A smaller amount of saturated acids (15–27%) is contained in the fat of farm-raised salmon. This is a consequence of the continuous development of fish farming in recent decades, which has led to fundamental changes in the composition of fish feed [1,11,15,18]. The linoleic acid content in wild fish is usually in the order of a few percent of fatty acids, while in salmon it reaches up to 15%. It is well known that linoleic acid is present in large quantities in most vegetable oils. Therefore, its intake from fish is completely marginal from a nutritional point of view. α-linolenic acid, which is relatively significant in the fatty acid profile of sockeye salmon and salmon samples (Table 3), is a precursor to the formation of biologically active EPA and DHA. Nutritional guidelines for the intake of fatty acids are given either in mass units or in % of the energy received—even in this case, however, the data must be converted to mass units. For the intake of α-linolenic acid, the nutritional guideline is 0.5% of the energy intake (EFSA, 2017 [2]). With an energy intake of 9300 kJ and 7500 kJ (see above), the required intake of α-linolenic acid is 1.26 g and 1.0 g. To assess the contribution of a given fat to fulfilling the nutritional guideline, the fatty acid content must also be converted to a weight unit. In this case, the fact is used that in common food lipids, fatty acids represent approximately 90% of the weight of the fat fraction [3,37,38]. The stated 5.6% α-linolenic acid in sockeye salmon represents 217 mg of α-linolenic acid in 100 g of product, 5.8% in salmon, and then 652 mg/100 g. Therefore, our results show that these two types of fish can contribute relatively significantly to the intake of insufficient α-linolenic acid. In the liver, dietary α-linolenic acid is metabolized by Δ5 and Δ6 desaturase enzymes to form EPA and DHA. The physiological importance of these acids is absolutely fundamental, as they are direct precursors of tissue hormones, eicosanoids, and docosanoids. Another significant benefit of EPA and DHA intake is the highly positive effect on the solubility of blood lipids [2,3,9,17,35,36,39], and it may lower metabolic dysfunction-associated steatotic liver disease [40]. The mutual ratio of EPA/DHA is very different in different fish species, but it has not yet been proven that it affects the formation of eicosanoids in any way [3,9,15,17,23,35,36,39]. For this reason, the sum of both acids is usually used in nutritional assessment. EFSA (2017) [2] issued a recommendation for an EPA and DHA intake of 250 mg per day for an adult. This amount should be covered by intake from diet and supplements, with intake from a normal diet being preferred. High content of EPA + DHA was found in cod liver, mackerel, and sardines; medium levels in sockeye salmon and farmed salmon; and lower content in the very popular canned fish, skipjack tuna. Fatty acids, as well as other lipophilic components, accumulate in the body. For that reason, it is, of course, possible to consume the necessary amount of the product in longer time intervals. PUFAs are known to be highly oxylabile. The high content of PUFAs in samples of canned fish is proof that the canning technology is conducted very carefully, and during the technological processing, oxygen is practically completely removed from the canned food, and the extent of oxidation is negligible [12,20,21,22,23,24,25,26,41,42].

## 5. Conclusions

The study demonstrated that the composition of the macronutrients in commercially available canned marine fish differs significantly by species. Protein content in assessed muscle samples was mostly between 15 and 22 g/100 g (with the exception of cod liver ≈ 7.3 g/100 g), with a typical value for canned muscle tissue of 18–20 g/100 g. Protein variability was relatively low, whereas fat content showed considerable variation (approximately 0.5–15 g/100 g). The lowest fat content was found in skipjack tuna (<1 g/100 g); sockeye salmon exhibited low fat content (2.8–4.3 g/100 g); sardines had moderate fat (~9–10 g/100 g); and mackerel and farmed Atlantic salmon had higher fat (10–15 g/100 g). The proportion of PUFAs was mostly 40–50% of total fatty acids; a lower proportion was observed in salmon and skipjack tuna. Probably the most significant nutritional benefit of consuming marine fish is the intake of long-chain n-3 HUFAs (EPA and DHA). With the exception of skipjack tuna, all the monitored products were good sources of these acids. Considering a typical reference serving size of 85 g for canned fish, this portion corresponds to an intake of approximately 250 mg EPA and DHA for two days (canned Atlantic and sockeye salmon), five days (sardines), and nine days (mackerel). These results support including canned marine fish in the diet as a source of high-quality protein and n-3 HUFAs, while species choice substantially affects the energy and lipid composition of a portion.

## Figures and Tables

**Table 1:** List of samples of canned fish products from the Czech market.

Nr.	Description	Fish	Brand Name	Country of Origin	Place of Capture
T1, T2 *	Tuna in brine, chunks	Skipjack tuna, *Katsuwonus pelamis* L.	Giana	Spain	FAO 34, middle eastern Atlantic Ocean
T3, T4 *	Tuna in brine, chunks	Skipjack tuna, *Katsuwonus pelamis* L.	Sun&Sea	Spain	FAO 34, middle eastern Atlantic Ocean
T5, T6 *	Tuna in brine, chunks	Skipjack tuna, *Katsuwonus pelamis* L.	Blue Bay	Vietnam	FAO 71, central Pacific Ocean
T7, T8, T9 *	Tuna in brine, chunks	Skipjack tuna, *Katsuwonus pelamis* L.	Rio Mare	Mauritius	FAO 51, Indian Ocean
T10, T11 *	Tuna in brine, chunks	Skipjack tuna, *Katsuwonus pelamis* L.	Wellness Nekton	Vietnam	FAO 71, central Pacific Ocean
T12, T13 *	Tuna in brine, chunks	Skipjack tuna, *Katsuwonus pelamis* L.	Giana	Ecuador	FAO 77,87 middle western Pacific Ocean
CL1–CL4 *	Cod liver in its own oil	Atlantic cod, *Gadus morhua* L.	Giana	Iceland	FAO 27, northeastern Atlantic Ocean
S1–S5 *	Salmon in brine, chunks	Atlantic salmon, *Salmo salar* L.	Nekton	Poland	Norway, farm breeding
O1, O2 *	Salted salmon nerka from Alaska	Sockeye salmon, *Oncorhynchus nerka* Walb.	Marks & Spencer	USA	FAO 67, north Pacific Ocean
O3, O4 *	Salmon nerka in salted brine	Sockeye salmon, *Oncorhynchus nerka* Walb.	Marks & Spencer	Canada	FAO 67, north Pacific Ocean
M1, M2 *	Mackerel in brine	Atlantic mackerel, *Scomber scombrus* L.	Giana	Morocco	FAO 34, middle eastern Atlantic Ocean
M3, M4 *	Mackerel in brine	Atlantic mackerel, *Scomber scombrus* L.	Nekton	Morocco	FAO 34, middle eastern Atlantic Ocean
M5, M6 *	Mackerel in brine	Atlantic mackerel, *Scomber scombrus* L.	Nekton	Latvia	FAO 34, middle eastern Atlantic Ocean
P1–P4 *	Sardines in brine	European pilchard, *Sardina pilchardus* Walb.	Giana	Morocco	FAO 34, middle eastern Atlantic Ocean
P5, P6 *	Sardines in brine	European pilchard, *Sardina pilchardus* Walb.	Tesco	Morocco	FAO 34, middle eastern Atlantic Ocean
P7	Sardines in brine	European pilchard, *Sardina pilchardus* Walb.	Gold-Plus	Morocco	FAO 34, middle eastern Atlantic Ocean
P8, P9 *	Sardines in brine	European pilchard, *Sardina pilchardus* Walb.	Hamé	Morocco	FAO 34, middle eastern Atlantic Ocean
P10–P12 *	Sardines in brine	European pilchard, *Sardina pilchardus* Walb.	Nekton Jardan	Morocco	FAO 34, middle eastern Atlantic Ocean
P13, P14 *	Sardines in brine	European pilchard, *Sardina pilchardus* Walb.	Giana	Morocco	FAO 34, middle eastern Atlantic Ocean

* Different batches.

**Table 2:** Proximate composition of the tested canned fish samples.

Sample				Sample			
No.	DM	Protein	Fat	No.	DM	Protein	Fat
	(g/100 g)	(g/100 g)	(g/100 g)		(g/100 g)	(g/100 g)	(g/100 g)
T1	18.9 ± 1.1	16.9 ± 0.8	0.5 ± 0.1	S1	37.0 ± 1.4	17.0 ± 1.2	12.4 ± 0.7
T2	18.9 ± 0.8	17.6 ± 1.4	0.8 ± 0.2	S2	38.8 ± 2.8	18.9 ± 1.0	12.6 ± 0.7
T3	17.8 ± 0.7	15.5 ± 0.6	0.8 ± 0.1	S3	39.5 ± 2.7	19.5 ± 1.0	12.5 ± 1.1
T4	19.6 ± 0.7	18.1 ± 1.1	0.6 ± 0.1	S4	38.7 ± 2.1	15.7 ± 0.7	11.5 ± 0.8
T5	20.7 ± 1.1	18.5 ± 0.9	0.6 ± 0.2	S5	40.6 ± 3.4	17.9 ± 0.8	12.0 ± 0.6
T6	19.9 ± 0.9	18.9 ± 0.9	0.7 ± 0.3	O1	29.8 ± 1.9	20.4 ± 0.9	4.3 ± 0.4
T7	20.8 ± 1.1	18.4 ± 0.6	0.5 ± 0.1	O2	28.5 ± 1.2	21.5 ± 0.8	3.9 ± 0.3
T8	20.1 ± 0.7	18.9 ± 1.1	0.6 ± 0.2	O3	24.6 ± 1.3	20.9 ± 1.0	3.2 ± 0.2
T9	19.9 ± 1.0	18.2 ± 1.0	0.9 ± 0.1	O4	26.3 ± 1.6	22.2 ± 0.9	2.8 ± 0.4
T10	22.0 ± 1.2	21.3 ± 1.0	0.4 ± 0.2	SP1	30.5 ± 1.2	14.7 ± 1.2	11.8 ± 0.8
T11	21.6 ± 1.0	20.6 ± 0.9	0.5 ± 0.1	SP2	29.9 ± 1.5	16.9 ± 1.0	10.6 ± 0.7
T12	21.5 ± 0.7	20.5 ± 0.5	0.6 ± 0.1	SP3	27.1 ± 1.6	18.7 ± 0.9	8.7 ± 0.8
T13	20.9 ± 1.0	19.8 ± 0.9	0.7 ± 0.3	SP4	28.6 ± 1.8	19.5 ± 1.0	7.9 ± 0.6
CL1	71.8 ± 3.2	7.2 ± 0.4	56.9 ± 3.4	SP5	29.2 ± 1.2	20.0 ± 0.8	7.3 ± 0.5
CL2	72.6 ± 3.8	6.8 ± 0.5	60.3 ± 2.2	SP6	28.9 ± 1.7	19.7 ± 1.1	7.6 ± 1.0
CL3	70.3 ± 3.5	6.8 ± 0.5	58.3 ± 3.0	SP7	32.2 ± 1.6	20.5 ± 0.6	8.6 ± 0.9
CL4	69.8 ± 4.5	8.3 ± 0.7	55.9 ± 3.8	SP8	32.1 ± 1.3	20.5 ± 0.9	10.4 ± 0.8
M1	30.7 ± 1.9	19.5 ± 0.8	10.3 ± 0.9	SP9	31.6 ± 1.4	20.8 ± 1.1	9.6 ± 0.5
M2	34.1 ± 2.4	20.3 ± 0.9	12.5 ± 0.7	SP10	32.1 ± 1.2	21.9 ± 1.1	10.0 ± 0.7
M3	32.9 ± 2.0	18.2 ± 1.1	11.9 ± 1.0	SP11	30.1 ± 1.3	19.7 ± 0.8	8.9 ± 0.4
M4	31.9 ± 2.1	19.1 ± 0.7	11.6 ± 0.7	SP12	31.0 ± 1.2	19.6 ± 0.9	10.1 ± 1.0
M5	36.2 ± 2.0	18.9 ± 1.2	16.2 ± 1.3	SP13	30.6 ± 1.3	21.8 ± 1.6	8.4 ± 0.6
M6	35.7 ± 1.5	19.5 ± 0.8	14.8 ± 0.5	SP14	30.9 ± 0.7	20.1 ± 0.7	9.4 ± 0.7

Legend: DM = dry matter content; Protein = crude protein content; Fat = fat content; Sample groups: T = tuna (skipjack); CL = cod liver; M = mackerel; S = salmon; O = salmon nerka; SP = sardine samples.

**Table 3:** Composition of selected fatty acids in canned fish products (as % of total fatty acids).

Sample No.	C14:0	C16:0	C18:1	C18:2	C18:3	C20:5	C22:6	C20:4
T1	14.3 ± 0.2	18.4 ± 0.3	12.8 ± 0.1	1.4 ± 0.1	0.4 ± 0.1	3.1 ± 0.1	11.7 ± 0.3	2.3 ± 0.1
T2	11.5 ± 0.1	23.6 ± 0.2	12.5 ± 0.1	1.1 ± 0.1	2.3 ± 0.1	2.0 ± 0.1	9.9 ± 0.2	1.2 ± 0.1
T3	18.4 ± 0.2	19.3 ± 0.3	11.4 ± 0.1	1.5 ± 0.1	0.9 ± 0.1	3.6 ± 0.1	6.3 ± 0.2	2.8 ± 0.1
T4	16.8 ± 0.1	16.8 ± 0.3	12.4 ± 0.2	1.3 ± 0.1	1.2 ± 0.1	3.8 ± 0.1	10.3 ± 0.3	2.5 ± 0.1
T5	18.3 ± 0.2	21.4 ± 0.3	11.8 ± 0.1	1.1 ± 0.1	0.3 ± 0.1	2.8 ± 0.1	12.8 ± 0.3	1.3 ± 0.1
T6	11.4 ± 0.1	21.7 ± 0.4	15.1 ± 0.1	1.9 ± 0.1	3.3 ± 0.1	2.5 ± 0.1	9.3 ± 0.3	1.6 ± 0.1
T7	17.7 ± 0.3	23.2 ± 0.4	12.0 ± 0.1	0.7 ± 0.1	0.2 ± 0.1	2.3 ± 0.1	9.0 ± 0.3	2.3 ± 0.1
T8	8.7 ± 0.1	21.3 ± 0.3	14.8 ± 0.2	0.8 ± 0.1	0.5 ± 0.1	2.9 ± 0.1	9.9 ± 0.2	1.5 ± 0.1
T9	11.0 ± 0.1	28.9 ± 0.3	10.9 ± 0.1	1.0 ± 0.1	1.8 ± 0.1	2.1 ± 0.1	8.7 ± 02	0.7 ± 0.1
T10	22.4 ± 0.3	15.6 ± 0.2	14.0 ± 0.3	0.7 ± 0.1	2.5 ± 0.1	2.6 ± 0.1	7.3 ± 0.1	2.1 ± 0.1
T11	12.3 ± 0.1	24.3 ± 0.3	13.3 ± 0.1	0.9 ± 0.1	0.9 ± 0.1	2.3 ± 0.1	9.0 ± 0.2	0.4 ± 0.1
T12	21.0 ± 0.2	16.7 ± 0.2	14.9 ± 0.2	0.7 ± 0.1	0.6 ± 0.1	2.2 ± 0.1	9.2 ± 0.2	0.3 ± 0.1
T13	17.2 ± 0.1	22.6 ± 0.2	12.3 ± 0.2	1.2 ± 0.1	1.2 ± 0.1	2.5 ± 0.1	8.9 ± 0.2	2.2 ± 0.1
CL1	5.6 ± 0.1	12.6 ± 0.1	17.4 ± 0.3	1.6 ± 0.1	9.3 ± 0.1	9.0 ± 0.2	10.5 ± 0.3	7.5 ± 0.1
CL2	6.0 ± 0.1	10.8 ± 0.1	15.9 ± 0.1	1.4 ± 0.1	8.8 ± 0.1	10.1 ± 0.2	9.8 ± 0.2	8.9 ± 0.1
CL3	4.6 ± 0.1	10.1 ± 0.1	19.6 ± 0.2	1.6 ± 0.1	13.6 ± 0.2	7.6 ± 0.1	10.3 ± 0.2	8.3 ± 0.1
CL4	5.0 ± 0.1	9.8 ± 0.1	21.3 ± 0.2	1.1 ± 0.1	11.2 ± 0.2	7.1 ± 0.1	9.5 ± 0.2	9.8 ± 0.2
M1	8.3 ± 0.2	18.2 ± 0.3	7.4 ± 0.1	2.5 ± 0.1	1.0 ± 0.1	13.0 ± 0.2	16.7 ± 0.3	3.1 ± 0.1
M2	6.8 ± 0.1	16.4 ± 0.1	8.6 ± 0.1	2.2 ± 0.1	1.3 ± 0.1	11.3 ± 0.2	18.1 ± 0.3	3.9 ± 0.1
M3	7.6 ± 0.1	17.1 ± 0.1	6.8 ± 0.1	2.1 ± 0.1	0.8 ± 0.1	11.2 ± 0.2	21.9 ± 0.4	3.1 ± 0.1
M4	7.5 ± 0.1	18.6 ± 0.3	7.9 ± 0.1	2.0 ± 0.1	1.1 ± 0.1	11.8 ± 0.2	17.5 ± 0.3	3.4 ± 0.1
M5	7.7 ± 0.1	15.5 ± 0.2	9.5 ± 0.2	2.9 ± 0.1	1.9 ± 0.1	10.0 ± 0.2	16.8 ± 0.3	4.3 ± 0.1
M6	8.0 ± 0.2	18.2 ± 0.3	10.1 ± 0.2	2.2 ± 0.1	1.6 ± 0.1	9.6 ± 0.1	17.3 ± 0.1	3.1 ± 0.1
S1	3.5 ± 0.1	9.1 ± 0.1	39.2 ± 0.3	14.9 ± 0.2	3.6 ± 0.1	2.7 ± 0.1	3.4 ± 0.1	1.7 ± 0.1
S2	7.2 ± 0.1	11.6 ± 0.2	36.5 ± 0.4	13.1 ± 0.2	4.2 ± 0.1	1.9 ± 0.1	5.6 ± 0.1	1.9 ± 0.1
S3	5.6 ± 0.1	8.8 ± 0.1	31.6 ± 0.4	15.4 ± 0.1	5.6 ± 0.1	2.9 ± 0.1	5.1 ± 0.1	0.9 ± 0.1
S4	9.0 ± 0.2	12.9 ± 0.2	33.3 ± 0.4	12.4 ± 0.2	5.8 ± 0.1	2.3 ± 0.1	5.0 ± 0.1	1.0 ± 0.1
S5	8.2 ± 0.1	11.9 ± 0.3	31.2 ± 0.3	14.6 ± 0.3	5.0 ± 0.1	2.8 ± 0.1	4.4 ± 0.1	1.5 ± 0.1
O1	7.6 ± 0.1	19.7 ± 0.3	11.9 ± 0.2	2.2 ± 0.1	5.6 ± 0.1	7.1 ± 0.1	19.7 ± 0.3	4.3 ± 0.1
O2	7.2 ± 0.1	18.8 ± 0.2	12.9 ± 0.1	2.0 ± 0.1	4.1 ± 0.1	7.8 ± 0.2	21.5 ± 0.4	4.0 ± 0.1
O3	5.3 ± 0.1	20.5 ± 0.3	13.2 ± 0.1	2.5 ± 0.1	3.8 ± 0.1	8.0 ± 02	20.5 ± 0.4	2.5 ± 0.1
O4	6.4 ± 0.1	18.5 ± 0.2	11.8 ± 0.1	2.3 ± 0.1	3.2 ± 0.1	7.2 ± 0.1	18.9 ± 0.3	3.1 ± 0.1
SP1	8.7 ± 0.1	19.1 ± 0.2	10.7 ± 0.1	3.5 ± 0.1	1.7 ± 0.1	20.1 ± 0.3	4.5 ± 0.1	1.4 ± 0.1
SP2	8.2 ± 0.1	18.9 ± 0.3	10.3 ± 0.2	3.1 ± 0.1	1.3 ± 0.1	19.3 ± 0.2	6.9 ± 0.1	1.0 ± 0.1
SP3	12.3 ± 0.3	23.6 ± 0.3	9.4 ± 0.1	3.0 ± 0.1	1.2 ± 0.1	15.7 ± 0.2	3.9 ± 0.1	1.4 ± 0.1
SP4	8.1 ± 0.1	19.8 ± 0.2	10.1 ± 0.2	2.8 ± 0.1	1.0 ± 0.1	16.8 ± 0.2	7.8 ± 0.1	0.9 ± 0.1
SP5	6.3 ± 0.1	17.6 ± 0.3	5.5 ± 0.1	3.7 ± 0.1	0.6 ± 0.1	25.4 ± 0.3	7.9 ± 0.2	1.3 ± 0.1
SP6	8.6 ± 0.2	17.9 ± 0.3	9.2 ± 0.1	2.5 ± 0.1	0.9 ± 0.1	18.6 ± 0.2	9.7 ± 0.1	1.1 ± 0.1
SP7	7.1 ± 0.1	19.9 ± 0.3	8.4 ± 0.2	1.6 ± 0.1	0.5 ± 0.1	15.5 ± 0.2	13.0 ± 0.3	1.6 ± 0.1
SP8	9.4 ± 0.2	19.6 ± 0.3	5.0 ± 0.1	2.7 ± 0.1	0.6 ± 0.1	20.4 ± 0.3	8.6 ± 0.2	0.7 ± 0.1
SP9	8.0 ± 0.2	18.8 ± 0.2	8.5 ± 0.2	2.0 ± 0.1	0.9 ± 0.1	18.6 ± 0.2	9.1 ± 0.2	1.2 ± 0.1
SP10	8.8 ± 0.1	20.8 ± 0.2	9.6 ± 0.2	3.2 ± 0.1	0.6 ± 0.1	19.7 ± 0.3	6.3 ± 0.1	0.2 ± 0.1
SP11	7.6 ± 0.1	19.5 ± 0.3	10.3 ± 0.3	2.6 ± 0.1	1.1 ± 0.1	17.9 ± 0.2	7.8 ± 0.1	0.9 ± 0.1
SP12	9.1 ± 0.1	18.9 ± 0.2	9.1 ± 0.2	2.8 ± 0.1	0.8 ± 0.1	18.2 ± 0.3	8.3 ± 0.1	0.5 ± 0.1
SP13	10.2 ± 0.3	20.9 ± 0.3	5.2 ± 0.1	2.9 ± 0.1	0.7 ± 0.1	21.1 ± 0.3	8.1 ± 0.2	0.7 ± 0.1
SP14	7.9 ± 0.1	18.9 ± 0.3	9.6 ± 0.1	2.5 ± 0.1	1.1 ± 0.1	18.9 ± 0.2	8.9 ± 0.1	0.6 ± 0.1

Values are expressed as mean ± standard deviation (SD); fatty acid: C14:0 = myristic acid; C16:0 = palmitic acid; C18:1 = oleic acid; C18:2 = linoleic acid; C18:3 = α-linolenic acid; C20:5 = eicosapentaenoic acid (EPA); C22:6 = docosahexaenoic acid (DHA); C20:4 = arachidonic acid; Sample groups: T = tuna (skipjack); CL = cod liver; M = mackerel; S = salmon; O = salmon nerka; SP = sardine samples.

**Table 4:** Composition of fatty acids in fish products (as % of total fatty acids and in mg/100 g of sample).

Sample No.	Σ SFA (%)	Σ SFA (mg/100 g)	Σ MUFA (%)	Σ MUFA (mg/100 g)	Σ PUFA (%)	Σ PUFA (mg/100 g)
T1	42.3 ± 0.4	190	20.6 ± 0.4	93	36.2 ± 0.4	163
T2	47.6 ± 0.4	343	18.8 ± 0.2	135	33.0 ± 0.3	238
T3	51.1 ± 0.5	368	17.8 ± 0.3	128	30.4 ± 0.3	219
T4	46.1 ± 0.4	332	18.9 ± 0.3	136	34.1 ± 0.3	246
T5	51.3 ± 0.5	277	17.8 ± 0.2	96	30.6 ± 0.3	165
T6	44.6 ± 0.4	281	23.9 ± 0.3	151	30.9 ± 0.3	195
T7	55.2 ± 0.5	248	17.9 ± 0.2	81	26.4 ± 0.2	119
T8	40.0 ± 0.4	216	25.9 ± 0.3	140	33.3 ± 0.3	180
T9	53.2 ± 0.5	431	16.9 ± 0.2	137	29.7 ± 0.3	241
T10	51.3 ± 0.5	185	22.2 ± 0.2	80	26.1 ± 0.2	94
T11	48.8 ± 0.4	220	20.6 ± 0.2	93	30.2 ± 0.3	136
T12	50.3 ± 0.5	272	22.6 ± 0.3	122	26.9 ± 0.3	145
T13	52.8 ± 0.5	333	18.9 ± 0.2	119	27.6 ± 0.3	174
CL1	21.3 ± 0.3	10,900	34.0 ± 0.4	17,400	44.4 ± 0.3	22,700
CL2	19.2 ± 0.3	10,400	27.5 ± 0.3	14,900	52.9 ± 0.4	28,700
CL3	17.7 ± 0.3	9290	35.6 ± 0.3	18,700	46.4 ± 0.4	24,300
CL4	20.5 ± 0.3	10,300	29.8 ± 0.3	15,000	49.5 ± 0.4	24,900
M1	35.0 ± 0.4	3250	18.7 ± 0.2	1730	46.0 ± 0.4	4260
M2	32.6 ± 0.3	3670	17.3 ± 0.2	1950	49.7 ± 0.5	5590
M3	34.5 ± 0.3	3700	17.8 ± 0.1	1910	47.6 ± 0.4	5100
M4	33.1 ± 0.3	3460	18.8 ± 0.3	1960	47.5 ± 0.4	4960
M5	28.6 ± 0.3	4170	23.0 ± 0.2	3350	48.3 ± 0.4	7040
M6	29.8 ± 0.3	3970	23.8 ± 0.3	3170	46.0 ± 0.4	6130
S1	15.3 ± 0.2	1710	52.7 ± 0.5	5880	31.7 ± 0.3	3540
S2	21.5 ± 0.2	2440	48.6 ± 0.5	5510	28.9 ± 0.3	3280
S3	19.4 ± 0.2	2180	41.5 ± 0.3	4670	38.8 ± 0.3	4370
S4	27.2 ± 0.3	2820	41.5 ± 0.4	4300	31.0 ± 0.3	3210
S5	25.3 ± 0.3	2730	39.8 ± 0.4	4300	33.9 ± 0.3	3660
O1	32.6 ± 0.3	1260	22.2 ± 0.1	859	45.1 ± 0.4	1750
O2	30.9 ± 0.3	1090	23.1 ± 0.2	811	44.9 ± 0.3	1580
O3	30.6 ± 0.3	881	24.6 ± 0.2	708	44.2 ± 0.3	1270
O4	29.8 ± 0.3	751	21.2 ± 0.3	534	48.2 ± 0.5	1220
SP1	32.2 ± 0.3	3420	26.2 ± 0.3	2780	40.9 ± 0.4	4340
SP2	30.8 ± 0.3	2940	23.8 ± 0.2	2270	45.0 ± 0.4	4290
SP3	41.5 ± 0.3	3250	25.9 ± 0.3	2030	32.1 ± 0.3	2510
SP4	33.8 ± 0.3	2400	24.6 ± 0.3	1750	41.1 ± 0.3	2920
SP5	28.9 ± 0.3	1900	22.6 ± 0.3	1490	48.2 ± 0.4	3170
SP6	30.1 ± 0.3	2060	19.5 ± 0.3	1330	49.8 ± 0.4	3410
SP7	33.6 ± 0.3	2600	25.0 ± 0.3	1940	41.0 ± 0.4	3170
SP8	33.9 ± 0.3	3170	20.5 ± 0.3	1920	45.1 ± 0.5	4220
SP9	30.9 ± 0.3	2670	19.9 ± 0.2	1720	48.6 ± 0.4	4200
SP10	35.0 ± 0.4	3150	25.7 ± 0.3	2310	38.6 ± 0.3	3470
SP11	33.5 ± 0.3	2680	20.6 ± 0.2	1650	45.3 ± 0.3	3630
SP12	33.3 ± 0.3	3030	16.9 ± 0.2	1540	49.1 ± 0.4	4460
SP13	36.6 ± 0.4	2770	20.8 ± 0.3	1570	42.2 ± 0.4	3190
SP14	31.8 ± 0.3	2690	20.9 ± 0.3	1770	47.0 ± 0.4	3980

Values are expressed as mean ± standard deviation (SD); fatty acid: SFA = saturated fatty acids; MUFA = monounsaturated fatty acids; PUFA = polyunsaturated fatty acids; Sample groups: T = tuna (skipjack); CL = cod liver; M = mackerel; S = salmon; O = salmon nerka; SP = sardine samples.

**Table 5:** Nutritional evaluation of canned fish in terms of content and intake of EPA and DHA.

Sample			Sample		
No.	Σ EPA, DHA	Amount of Sample for 250 mg of EPA + DHA	No.	Σ EPA, DHA	Amount of Sample for 250 mg of EPA + DHA
	(mg/100 g of Sample)	(g)		(mg/100 g of Sample)	(g)
T1	66.6	375	S1	680.8	36.7
T2	85.7	292	S2	850.5	29.4
T3	71.3	351	S3	900.0	27.8
T4	76.1	328	S4	755.6	33.1
T5	84.2	297	S5	777.6	32.2
T6	74.3	336	O1	1037	24.1
T7	50.9	492	O2	1028	24.3
T8	69.1	362	O3	820.8	30.5
T9	87.5	286	O4	657.7	38.0
T10	35.6	702	SP1	2613	9.6
T11	50.9	492	SP2	2500	10.0
T12	61.6	406	SP3	1535	16.3
T13	71.8	348	SP4	1749	14.3
CL1	9986	2.5	SP5	2188	11.4
CL2	10,800	2.3	SP6	1936	12.9
CL3	9392	2.7	SP7	2206	11.3
CL4	8352	3.0	SP8	2714	9.2
M1	2753	9.1	SP9	2393	10.4
M2	3308	7.6	SP10	2340	10.7
M3	3545	7.1	SP11	2059	12.1
M4	3058	8.2	SP12	2409	10.4
M5	3907	6.4	SP13	2208	11.3
M6	3583	7.0	SP14	2352	10.6

Sample groups: T = tuna (skipjack); CL = cod liver; M = mackerel; S = salmon; O = salmon nerka; SP = sardine samples.

## Data Availability

The data analyzed during the current study are available from the corresponding author upon reasonable request.

## References

[1] Pohorela B., Gramblicka T., Dolezal M., Dvorakova D., Pulkrabova J., Kourimska L., Ilko V., Panek J. (2022). Nutritional Quality and Assessment of Contaminants in Farmed Atlantic Salmon (*Salmo salar* L.) of Different Origins. J. Food Qual..

[2] EFSA (2017). Dietary Reference Values for Nutrients. Summary Report. European Food Safety Authority. EFSA Tech. Rep..

[3] Velisek J. (2014). The Chemistry of Food.

[4] Souci S.W., Fachmann W., Kraut H. (2008). Food Composition and Nutrition Tables.

[5] Sikorski Z.E., Kolakowska A., Sun Pan B., Sikorski Z.E. (1990). The Nutritive Composition of the Major Groups of Marin Food Organisms. Seafood: Resources, Nutritional Composition and Preservation.

[6] USDA Database: U.S. Department of Agriculture, Agricultural Research Services, 2024. https://fdc.nal.usda.gov/.

[7] Simat V., Hamed I., Petricevic S., Bogdanovic T. (2020). Seasonal Changes in Free Amino Acid and Fatty Acid Compositions of Sardines, *Sardina pilchardus* (Walbaum, 1792): Implications for Nutrition. Foods.

[8] Santarpia L., Contaldo F., Pasanisi F. (2017). Dietary protein content for an optimal diet: A clinical view. J. Cachexia Sarcopenia Muscle.

[9] Tilami S.K., Sampels S. (2017). Nutritional Value of Fish: Lipids, Proteins, Vitamins, and Minerals. Rev. Fish. Sci. Aquac..

[10] Layman D.K., Anthony T.G., Rasmussen B.B., Adams S.H., Lynch C.J., Brinkworth G.D., Davis T.A. (2015). Defining meal requirements for protein to optimize metabolic roles of amino acids. Am. J. Clin. Nutr..

[11] Berge G.M., Krogdahl A., Hillestad M., Holm H., Holm J., Ruyter B. (2023). Comparison of EPA and DHA utilization in Atlantic salmon (*Salmo salar*) and rainbow trout (*Oncorhynchus mykiss*) fed two diets with different content of fish oil and rapeseed oil. J. Appl. Aquac..

[12] Nava V., Turco V.L., Licata P., Panayotova V., Peycheva K., Fazio F., Rando R., Di Bella G., Potortì A.G. (2023). Determination of Fatty Acid Profile in Processed Fish and Shellfish Foods. Foods.

[13] Hu L., Hu Z., Chin Y., Yu H., Xu J., Zhou J., Liu D., Kang M., Hu Y. (2022). Lipidomic profiling of Skipjack tuna (*Katsuwonus pelamis*) by ultrahigh-performance liquid chromatography coupled to high resolution mass spectrometry. Fish. Aquat. Sci..

[14] Devadawson C. (2021). Effects of Different Type of Processing Methods on Biochemical Changes of Skipjack Tuna *Katsuwonus pelamis*. Int. J. Sci. Res..

[15] Sprague M., Fawcett S., Betancor M.B., Struthers W., Tocher D.R. (2020). Variation in the nutritional composition of farmed Atlantic salmon (*Salmo salar* L.) fillets with emphasis on EPA and DHA contents. J. Food Compos. Anal..

[16] Ravnskov U., DiNicolantonio J.J., Harcombe Z., Kummerow F.A., Okuyama H., Worm N. (2014). The Questionable Benefits of Exchanging Saturated Fat with Polyunsaturated Fat. Mayo Clin. Proc..

[17] FAO (2010). Fats and fatty acids in human nutrition. Report of an expert consultation. FAO Food and Nutrition Paper 2010.

[18] Blanchet C., Lucas M., Julien P., Morin R., Gingras S., Dewailly E. (2005). Fatty Acid Composition of Wild and Farmed Atlantic Salmon (*Salmo salar*) and Rainbow Trout (*Oncorhynchus mykiss*). Lipids.

[19] Bandarra N.M., Batista I., Nunes J.M., Empis J.M., Christie W.W. (1997). Seasonal Changes in Lipid Composition of Sardine (*Sardina pilchardus*). J. Food Sci..

[20] Nartea A., Ismaiel L., Frapiccini E., Falcone P.M., Pacetti D., Frega N.G., Lucci P., Colella S. (2023). Impact of Modern Oven Treatments on Lipid Oxidation and Vitamin E Content of Fillets from Sardine (*Sardina pilchardus*) at Different Reproductive Cycle Phases. Antioxidants.

[21] Villamarín E., Martínez B., Trigo M., Aubourg S.P. (2023). Influence of Different Previous Frozen Holding Periods on the Canned Fish Quality. Foods.

[22] Pal J., Ravi O.P.K., Kumari S., Singh A.K. (2021). Preservation of Seafoods by Hurdle Technology. Meat & Nutrition.

[23] Singer P., Richter V., Singer K., Löhlein I. (2021). Analyses and Declarations of Omega-3 Fatty Acids in Canned Seafood May Help to Quantify Their Dietary Intake. Nutrients.

[24] El Lahamy A.A., Mohamed H.R. (2020). Changes in Fish Quality During Canning Process and Storage Period of Canned Fish Products: Review Article. Glob. J. Nutr. Food Sci..

[25] Augustin M.A., Riley M., Stockmann R., Bennett L., Kahl A., Lockett T., Osmond M., Sanguansri P., Stonehouse W., Zajac I. (2016). Role of food processing in food and nutrition security. Trends Food Sci. Technol..

[26] Bratt L. (2013). Technical Guide to Fish Canning.

[27] Mackie I.M., Pryde S.E., Gonzales-Sotelo C., Medina I., Peréz-Martín R., Quinteiro J., Rey-Mendez M., Rehbein H. (1999). Challenges in the identification of species of canned fish. Trends Food Sci. Technol..

[28] Mahaliyana A.S., Jinadasa B.K.K.K., Liyanage N.P.P., Jayasinghe G.D.T.M., Jayamanne S.C. (2015). Nutritional Composition of Skipjack Tuna (*Katsuwonus pelamis*) Caught from the Oceanic Waters around Sri Lankae. Am. J. Food Nutr..

[29] (2023). Meat and Meat Products—Determination of Moisture Content—Reference Method.

[30] Barbano D.M., Clark J.L., Dunham C.E., Flemin R.J. (1990). Kjeldahl Method for Determination of Total Nitrogen Content of Milk: Collaborative Study. J. Assoc. Off. Anal. Chem..

[31] (2011). Regulation (EU) No 1169/2011 of the European Parliament and of the Council of 25 October 2011 on the Provision of Food Information to Consumers. Current Consolidated Version: 01/01/2018. Eur-Lex Document 32011R1169. http://data.europa.eu/eli/reg/2011/1169/oj.

[32] (2009). Oilseeds—Determination of Oil Content (Reference Method).

[33] (2011). Animal and Vegetable Fats and Oils—Gas Chromatography of Fatty Acid Methyl Esters—Part 2: Preparation of Methyl Esters of Fatty Acids.

[34] Sabolová M., Johanidesová A., Hasalíková E., Fišnar J., Doležal M., Réblová Z. (2017). Relationship between the composition of fats and oils and their oxidative stability at different temperatures, determined using the Oxipres apparatus. Eur. J. Lipid Sci. Technol..

[35] Klurfeld D.M., Akoh C.C., Min D.B. (2008). Dietary Fats, Eicosanoids, and the Immune System. Food Lipids. Chemistry, Nutrition, and Biotechnology.

[36] Kritchevsky D., Akoh C.C., Min D.B. (2008). Fats and Oils in Human Health. Food Lipids. Chemistry, Nutrition, and Biotechnology.

[37] O’Keefe S.F., Akoh C.C., Min D.B. (2008). Nomenclature and Classification of Lipids. Food Lipids. Chemistry, Nutrition, and Biotechnology.

[38] Belitz H.D., Grosch W., Schieberle P. (2009). Food Chemistry.

[39] Chen J., Jayachandran M., Bai W., Xu B. (2022). A critical review on the health benefits of fish consumption and its bioactive constituents. Food Chem..

[40] Tatoli R., Caterina B., Donghia R., Pesole P.L., Fontana L., Giannelli G. (2025). Dietary Omega-3 Fatty Acids from Fish and Risk of Metabolic Dysfunction-Associated Steatotic Liver Disease in a Mediterranean Population: Findings from the NUTRIHEP Cohort. Nutrients.

[41] Prego R., Trigo M., Martínez B., Aubourg S.P. (2022). Effect of Previous Frozen Storage, Canning Process and Packing Medium on the Fatty Acid Composition of Canned Mackerel. Mar. Drugs.

[42] Kołakowska A., Stypko K., Domiszewski Z., Bienkiewicz G., Perkowska A., Witczak A. (2002). Canned cod liver as a source of n-3 polyunsaturated fatty acids, with a reference to contamination. Nahrung.

